# Age Determination of the Glassy-Winged Sharpshooter, *Homalodisca vitripennis,* using Wing Pigmentation

**DOI:** 10.1673/031.011.7801

**Published:** 2011-07-01

**Authors:** Chris Timmons, Aaron Hassell, Isabelle Lauziere, Blake Bextine

**Affiliations:** ^1^ University of Texas, Tyler, 3900 University Blvd., Tyler, TX 75799; ^2^ Texas AgriLife Research, 259 Business Court, Fredericksburg, TX 78624; ^3^ P.O. Box 61, Harper, TX 78631

**Keywords:** insect pigments, vector insects, insect age

## Abstract

A red pigment is contained in the wing veins of the glassy-winged sharpshooter, *Homalodisca vitripennis* (Hemiptera: Cicadellidae). This insect is the main vector of the plant-pathogenic bacterium *Xylella fastidiosa* Wells (Xanthomonadales: Xanthomonadaceae), the causal agent of Pierce's disease of grapevines. Over the course of the *H. vitripennis* lifespan, the red pigment darkens and eventually becomes brown/black in color. These pigments are believed to be pheomelanin and eumelanin, respectively. The age of *H. vitripennis* can be determined by calculating the amount of red pigment found in the wings by analyzing high resolution wing photographs with image analysis software. In this study, a standard curve for the age determination of *H. vitripennis* was developed using laboratory—reared insects of known ages varying from 1 to 60 days. The impact of three environmental conditions on these readings was also investigated and found to have little effect on the age determination, and could be easily accounted for. Finally, field collected insects from several Central Texas vineyards were successfully analyzed for age determination suggesting that the annually reported influx of *H. vitripennis* was composed almost entirely of older insects.

## Introduction

The glassy-winged sharpshooter, *Homalodisca vitripennis* (Hemiptera: Cicadellidae), is a xylem fluid-feeding insect native to the southwestern United States. In recent years, this insect has been well studied because of its role in the transmission of *Xylella fastidiosa* Wells (Xanthomonadales: Xanthomonadaceae), a pathogenic bacterium that causes Pierce's disease of grapevines (Goheen et al. 1973; Hopkins 1973; Michaei et al. 1978). This disease is the major limiting factor for grape production in Texas and California (Hopkins 1977). The glassy-winged sharpshooter gets its name for its mostly transparent forewings in the adult stage. The wing veins contain a distinctive red pigment of a brightness and presence that visibly fades with age. The vectoral capacity of these insects may differ according to age since it has been shown that *H. vitripennis* of differing ages have differing numbers of sensillia on their mouthparts (Leopold et al. 2003). This is of importance since *X. fastidiosa* usually accumulates in the mouthparts and foregut of the glassy-winged sharpshooter (Brlansky et al. 1983; Brlansky et al. 1982; Purcell et al. 1979).

Melanins, including pheomelanin and eumelanin, are ubiquitous in nature and are generally derivatives of the amino acid tyrosine. In insects and other arthropods, quinones and their derivatives have long been known to function in various melanization and sclerotization reactions associated with cuticle formation and repair (Brunet 1980). It has been found that the visible depigmentation of melanocytes does not require the degradation of existing melanin, but rather is due to the dilution of existing melanin by melanocyte turnover (Le Pape et al. 2008). The veins of the forewings of adult *H. vitripennis* have a distinctive red pigment, proposed to be pheomelanin, which can be converted into a dark brown/black pigment (probably eumelanin), as the insect ages. This change in wing vein pigmentation is a static change that is not altered postmortem (Bextine 2008). Since the pigmentation change observed in *H. vitripennis* seems to occur in correlation with age, analysis of the amount or concentration of one of these pigments could be used for age determination. An effective method of *H. vitripennis* age determination could be of great value when observing population dynamics and relationships of insects present in commercial vineyards. Accurate information on insect ages could then lead to a greater understanding of *H. vitripennis* pathogen transmission characteristics.

In order to monitor the presence of this insect in vineyards of California and Texas, *H. vitripennis* were collected using sticky card traps. This method works very well for monitoring population abundance and temporal distribution, and identifying species that occur at a given location. Such monitoring is important since this vector can disperse widely (Blua 2003), has a wide host range (Hoddle et al. 2003), and can cause spread of *X. fastidiosa* to new areas at an alarming rate (Redak et al. 2004). Determination of the age of *H. vitripennis* collected from these traps can be difficult due to the degradation of internal tissues. According to previous studies, the change in *H. vitripennis* forewing pigmentation was found to be linear in nature and is an effective method of age determination (Bextine 2008). However, the study was used only for 1- to 21-day old insects and many field—collected insects were found to be much older (Bextine 2008). Further research was needed in order to extend this correlation in order to obtain a more accurate linear regression equation with which to evaluate the ages of field—collected insects. Additionally, a more precise method of wing pigment evaluation was needed since the previous methods did not yield reliable and consistent enough results. In this study, a standard curve for age determination of *H. vitripennis* was developed, the effects of three environmental conditions on age determination were evaluated, and field collected insects from seven vineyards located near Fredericksburg, Texas during the timeframe of the annually reported influx of *H. vitripennis* were analyzed for age and any relationships that might exist between and within vineyards.

## Materials and Methods

### Preparation of a Standard Curve

**Figure 1.**
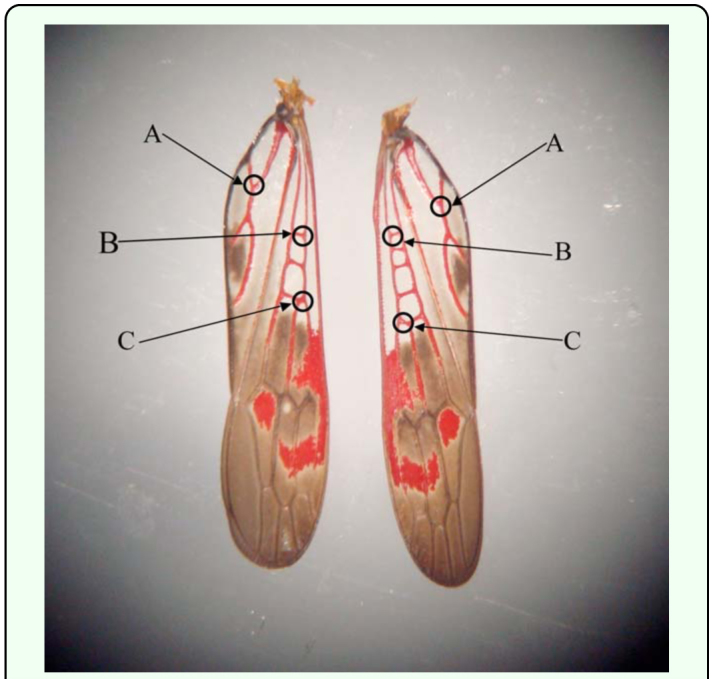
Red pigmentation analysis points. (A) Junction of the first branch of the cubitus vein, (B) most proximal junction of subcosta vein and subcosta-radial cross vein, and (C) junction of first branch of the subcosta vein. High quality figures are available online

Successful *H. vitripennis* age determination was accomplished by quantifying the color histogram values of high—resolution photographs of forewings removed from *H. vitripennis* of known ages. Wings from laboratory reared insects were removed and photographed through a dissecting microscope (Nikon SMZ-1, www.nikon.com), focused appropriately with a 7.2 mega pixel Sony digital camera. The ages of the insects analyzed were 1, 3, 6, 9 12, 15, 30, and 60 days after the molt to adult. Although *H. vitripennis* can live longer than 60 days, this was chosen as a starting point due to difficulty of successfully rearing insects in greenhouses for extended periods of time. If needed, older insects would be reared for successive assays. Ten *H. vitripennis* were used for each age observed. Red, blue, and green pixilation values from the color histogram of three specific sites on each forewing were observed (Figure 1). According to the Comstock-Needham insect wing venation naming system, the three sites from each forewing where data were collected were the most proximal junction of subcosta vein and subcosta-radial cross vein, the junction of first branch of the subcosta vein, and the junction of the first branch of the cubitus vein (Figure 1). At each data collection site, the data collected were where the blue and green values were the lowest within the area of the collection site in order to maintain consistency between collected data. The red pixilation values of the color histogram were then observed and recorded by using the Paint Shop Pro 7.02 image analysis software. The average of all three readings for each insect was then averaged with all other insects of the same age. A one-way ANOVA was performed to determine the effect of age on the average red pixilation values. For each age, the range and the standard deviation was also calculated. The results were then compiled into a standard curve.

### Impact of Environmental Conditions on Age Determination

In order to determine the consistency of age readings from field collected insects and laboratory reared insects, an additional experiment was designed. Laboratory reared insects were used for this experiment; these were killed by freezing and both forewings were removed. One wing from each insect was exposed to one of three environmental conditions to which *H. vitripennis* are commonly subjected to when caught on yellow sticky traps in the field, and one wing was used as a control. These three conditions were: 1) exposure to sun radiation for 1, 7, or 14 days inside of a plastic bag; 2) contact with the glue used on yellow sticky traps (accomplished by manually placing *H. vitripennis* on a trap and then removing); and 3) exposure to histo-clear (Histo-Clear II from National Diagnostics, www.nationaldiagnostics.com), an orange oil which is commonly used to dissolve the glue from *H. vitripennis* collected on sticky traps. Ten insects for the glue and hiso-clear tests were used while 60 insects were used for the sun exposure test (20 replicates each of 1, 7, and 14 days old). The wings submitted to the environmental conditions as well as the control wings from each insect were photographed and analyzed as previously described.

### Age Determination of Field Collected Insects

Glassy-winged sharpshooters were collected from seven vineyards in the areas surrounding Fredericksburg, Texas using Seabright Laboratories (www.seabrightlabs.com) sticky traps. Traps were set up in vineyards and then removed approximately two weeks later. All *H. vitripennis* caught on the traps were removed and labeled appropriately according to location and date (n = 50 insects). The time period of collection was between 26 May 2009 and 24 June 2009. This time period was used because this is when an “influx” of insects is known to occur consistently every year (Lauziere et al. 2008). The age of the insects were relevant because if they were young insects it would add weight to existing theories that F1 generation adults (F0 being the overwintering parents) have recently emerged and are actively searching suitable host plants. In two of the vineyards (labeled B and D), samples were collected approximately one month apart in order to compare the ages within the same vineyard. The wings of each *H. vitripennis* were removed and photographed as previously described.

**Figure 2.**
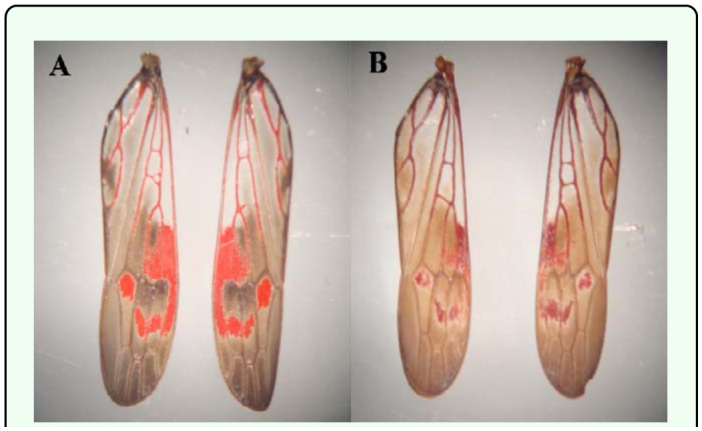
Comparison of wings from I-day-old adult *Homalodisca vitripennis* (A) and 60-day-old adult *H. vitripennis* (B). High quality figures are available online

## Results

### Preparation of a Standard Curve

**Figure 3.**
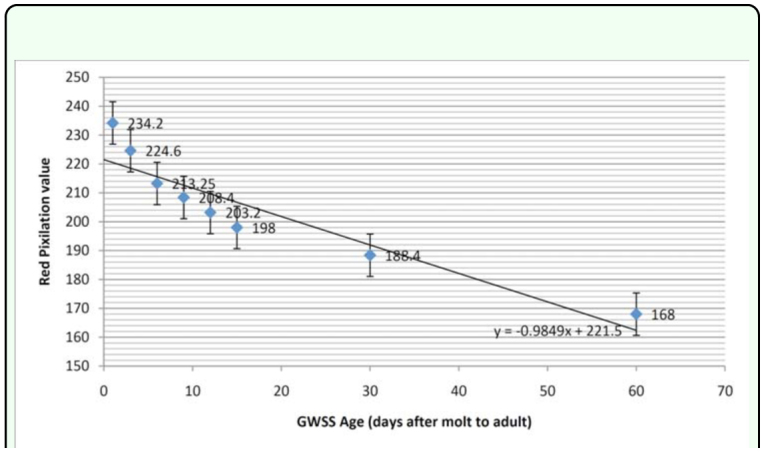
Red pixilation values from color histogram of high resolution wing photographs in relation to glassy winged sharpshooter age, showing a linear relationship. High quality figures are available online

The age of laboratory reared *H. vitripennis* was determined by pixilation analysis of high resolution forewing photographs. Adults of *H. vitripennis* of different ages were observed to have relatively diverse levels of red pigment in their wings that can be quantified and used for age determination. The characteristic red pigmentation observed in these insects decreased with age as it was converted to a darker brown/black pigment (Figure 2). The differences between readings of the two wings of a single insect were found to be non-significant (*p* > 0.05), and therefore only one wing was used for each insect. Using three data collection points corresponding to three different veins for each insect reduced the amount of variation between different parts of the wing. Most of the readings were within ten units of the average red pixilation value. The differences between the average readings of the ten insects of a given age were also usually relatively small with only one age group (12 days) having a standard deviation of greater than 10 (Table 1). The range of the standard deviations for each age was between 3.20 and 11.69. The significance level between each of the eight ages observed, except for between 9 and 12 days and 12 and 15 days, was 0.000 (Table 1). The p-values between 9 and 12 days and 12 and 15 days were 0.028 and 0.029, respectively. A linear relationship was found for red pixilation values of the color histogram from highmdash;resolution wing photographs plotted in relation to age (Figure 3). Linear regression of this data is represented by the equation y = -0.9849x + 221.5, where y is the red pixilation value and x is the insect age in days. One-way ANOVA shows that the standard curve has a confidence level of at least 97%.

**Table 1.**
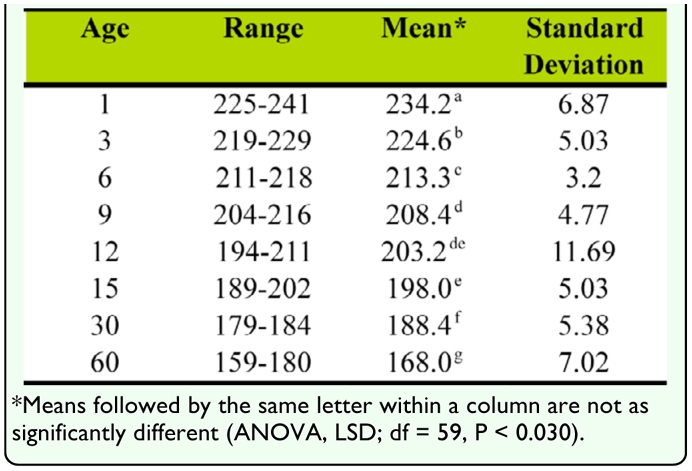
Range, mean, and standard deviation of red pixilation values from color histogram of wing photographs from insects of known ages used for age determination standard curve development.

### Impact of Environmental Conditions on Age Determination

Histo-clear and trap glue did not affect color reading of the wing, and therefore did not affect age determination (*p* > 0.05). The highest variation of red pixilation values between the control wing and experimental wing for insects submitted to glue and histoclear were 9 and 7, respectively. A paired t-test was used to calculate the statistical significance of the two experiments and yielded a *p* value of 0.1467 for the glue test and *a p* value of 0.1343 for the histo-clear test. These results express no statistical significance between the control and experimental wings. Exposure to sun radiation, on the other hand, had a much greater effect (*p* < 0.01). Sun exposure prematurely darkened the red pigments thus causing the calculated insect age to be greater than it actually was. A significant amount of variation was observed for each of the three ages tested. The difference in red pixilation values for the 1, 7, and 14 day old replicates were 0–17, 19–36, and 32–46 with averages of 9.45, 26.05, and 37.3, respectively. Due to the lack of a strong linear correlation with these results, it was not possible to determine how long an individual insect has been on a trap in the field with an acceptable level of accuracy. The average difference in red pixilation of all insects observed in this test was 24.27.

### Age Determination of Field Collected Insects

The ages of wild *H. vitripennis* adults trapped in seven commercial vineyards were determined in May and June 2009. The average age was 83.3 ± 16.32 days. In this assay, the effect of sun exposure on the wings of the insects was accounted for by adding 25 red pixilation units to the observed values. This number was used due to the average difference observed (24.27 red pixilation units) between experimental and control wings used for all insects in the sun exposure test (n = 60) since it is impossible to accurately quantify how long an insect has been on a trap in the field. The age of these insects were then calculated using the equation y = -0.9849x + 221.5. The range of ages observed was from 51.2 days to 112.1 days (Table 2). The youngest insect was collected on 26 May 2009 in vineyard E and the oldest one on 23 June 2009 in vineyard D. In vineyards B and D, two sampling dates were selected: 27 days apart in vineyard B and 28 days apart in vineyard D. The calculated average age difference between both sampling dates for vineyards B and D were 19 and 20 days, respectively.

**Table 2.**
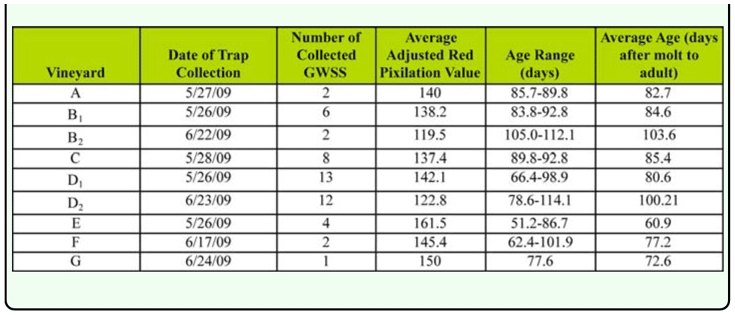
The calculated age from adjusted red pixilation values of 50 *Homalodisca vitripennis* collected from 9 vineyards between 26 May 2009 and 24 June 2009.

## Discussion

Age may be correlated to the vectoral capacity of *H. vitripennis* due to differing numbers of sensillia on the mouthparts of *H. vitripennis* of differing ages (Leupold et al. 2003). In order to determine if any such relationship exists, an effective method of age determination must be produced. As *H. vitripennis* ages, the amount of red pigment (probably pheomelanin) in their wings gradually decreases whereas the amount of brown/black pigment (probably eumelanin) gradually increases. Pheomelanin has been observed to be easily converted to eumelanin if the solution is lacking a high concentration of sulfur compounds (Shoshuke 1988). Therefore, if cysteine concentrations are low, pheomelanin will readily convert into eumelanin. In the case of *H. vitripennis,* the lack of cysteine would most likely come from a change in diet, which could have an effect on vectoral capacity and the likelihood of *H. vitripennis* feeding on *V. vinifera* grapevines. However, further research is needed to verify that the pigment is in fact pheomelanin. In this study, we showed the existence of a linear relationship between the age of adult *H. vitripennis* and the amount of red pigment in their forewings. A logarithmic curve fit the data slightly better, but was abandoned in favor of the linear curve due to the flattening out of the logarithmic curve with insects older than about 70 days, thus rendering the age determination of older insects ineffective. Thus, insects of ages older than about 70 days cannot be aged with an acceptable level of accuracy based on this model. However, population dynamics can still be successfully quantified by observing the relative ages of older insects. The change in forewing pigmentation observed in these insects can be fairly accurately quantified by analyzing the red pixilation of specific sites on excised forewings in high—resolution digital photographs. By developing a standard curve for *H. vitripennis* age using laboratory reared insects of known ages, the ages of field collected insects can be accurately evaluated up to about 70 days. This method of *H. vitripennis* age determination represents an easily reproducible, cost effective, accurate, and novel approach to insect age analysis. The data showed that each average red pixilation value for insects of known ages was statistically significant.

However, reared and wild *H. vitripennis* experience very different environmental conditions that could directly impact the accuracy of wing pigmentation evaluation based on the impacts these conditions have on melanins. Adults of *H. vitripennis* collected on sticky traps are submitted to two main conditions: sun radiation for a period of time varying from 1 to 14 days and the glue that covers the trap which is then dissolved in oil (orange oil in this case) in order to retrieve the specimen. In order to account for these conditions, reared insects were submitted to either sun radiation for one week on a trap placed inside a plastic bag or to orange oil for a few minutes the same way they would be in the extraction of a specimen from a trap. These assays indicated that glue and orange oil have no significant effect on wing pigmentation analysis, and therefore do not need to be accounted for when determining the age of wild *H.* *vitripennis.* Exposure to sun radiation, however, has a definite and significant impact on pigmentation analysis. It was found that the sun stimulated premature darkening of the pigments, thus giving the impression that collected insects are older than they really are. Therefore, when determining the age of field collected *H. vitripennis* from traps collected on a twomdash;week basis, one can add 25 to the calculated red pixilation value. This was determined by averaging all of 60 insects exposed to sun radiation over a two—week period. This adjusted value can then be inserted into the equation for the linear regression line (y = 0.9849x + 221.5, where x is age) in order to more precisely assess the age of wild insects.

In the Texas Hill Country grape growing regions, *H. vitripennis* adults are known to migrate from their overwintering hosts to grapevines from the last days of May to mid June every year (Lauziere et al. 2008). Until now it was believed that most of the adults that migrated were those of the first adult generation produced by the overwintering adults. The age of wild *H. vitripennis* caught in vineyards during the migration period was calculated by analysis of forewing pigmentation, taking into account the effects of sun exposure on wing pigmentation. It was observed that most of the insects were over 80 days old. The first egg masses of *H. vitripennis* are collected in mid February, with large amounts harvested in March (Lauziere unpublished observations). The F1 adults emerge by early April, thus allowing them up to 60 days to grow and then migrate as adults. On the other hand, overwintering adults emerge in October, offering the adults some 118 days to survive and then migrate. These data show that both overwintering adults and young adults migrate together to the vineyards when the grapevines are suitable. In two of the vineyards (B and D), samples were collected approximately one month apart in order to compare the ages within the same vineyard. The results showed that the average age between the two approximately one—month periods were between 19 and 20 days, thus exemplifying the relative accuracy of this method. Further research is needed to better understand the processes and cues involved in the migration of glassy-winged sharpshooters. Such data would help stimulate more effective management strategies in the future.
